# Studies of Turing pattern formation in zebrafish skin

**DOI:** 10.1098/rsta.2020.0274

**Published:** 2021-12-27

**Authors:** Shigeru Kondo, Masakatsu Watanabe, Seita Miyazawa

**Affiliations:** Graduate School of Frontier Biosciences, Osaka University, 1-3 Yamadaoka, Suita, Osaka 565-0871, Japan

**Keywords:** zebrafish, Turing pattern, pigment cells, reaction–diffusion, KT model, evolution

## Abstract

Skin patterns are the first example of the existence of Turing patterns in living organisms. Extensive research on zebrafish, a model organism with stripes on its skin, has revealed the principles of pattern formation at the molecular and cellular levels. Surprisingly, although the networks of cell–cell interactions have been observed to satisfy the ‘short-range activation and long-range inhibition’ prerequisites for Turing pattern formation, numerous individual reactions were not envisioned based on the classical reaction–diffusion model. For example, in real skin, it is not an alteration in concentrations of chemicals, but autonomous migration and proliferation of pigment cells that establish patterns, and cell–cell interactions are mediated via direct contact through cell protrusions. Therefore, the classical reaction–diffusion mechanism cannot be used as it is for modelling skin pattern formation. Various studies are underway to adapt mathematical models to the experimental findings on research into skin patterns, and the purpose of this review is to organize and present them. These novel theoretical methods could be applied to autonomous pattern formation phenomena other than skin patterns.

This article is part of the theme issue ‘Recent progress and open frontiers in Turing's theory of morphogenesis’.

## 1. Introduction

### (a) The importance of Turing's reaction–diffusion system in morphogenesis research

In the development of living organisms, a complex spatial order is automatically created from an egg with a simple structure. This is the most remarkable feature of life, and elucidating its principles is a major goal of embryological research. Turing's reaction–diffusion system [1] was first proposed as a principle to explain the autonomous nature of biological pattern formation. This theory is now widely accepted and applied in the studies of various pattern formation phenomena [2,3]. Turing's mathematical model was first published a long time ago (1952), but initially, the theory was not widely known. The theory was rediscovered about 20 years later by several mathematical biologists [4,5]. They used computer simulation and beautifully showed that the mathematical model can generate various spatial patterns of organisms [4,5]. However, many biologists did not immediately accept the idea, probably because of the lack of convincing experimental proof and the existence of another principle—the ‘positional information model’ or ‘morphogen gradient model’—that was widely accepted by biologists [6,7].

### (b) Positional information model

The positional information model or morphogen gradient model is a simple idea proposed by Wolpert around 1970 to explain morphogenesis during embryogenesis [6]. The model assumes that a diffusible molecule (morphogen) is localized in the region of the fertilized egg. As the molecule diffuses, a concentration gradient is formed, and each cell in the embryo can tell its own location according to the concentration of the molecule. By expressing a gene specific to that location, the cell can create a spatial pattern in the embryo. In the 1980s and 1990s, many papers were published proving the existence and function of morphogen molecules, and this idea became the standard for pattern formation principles in morphogenesis [3,8]. In fact, it has been demonstrated that most fertilized eggs contain morphogen-like molecules and that early development depends on the concentration of these molecules [8]. The positional information model is very flexible, and almost any positional information can be formed by manipulating the position of the morphogen source. For example, experiments have shown that repetitive patterns, such as the stripe pattern of segmented genes in *Drosophila*, which seem to be difficult to create with the morphogen gradient, can be created by combining multiple concentration gradients of morphogens [9,10]. This proof temporarily reduced the interest in reaction–diffusion systems among experimental biologists since the stripe expression pattern of segmental genes had earlier been claimed by some mathematicians to be evidence of a reaction–diffusion system at work.

### (c) Fish skin pattern as an experimental system to study the Turing pattern

Although the morphogen gradient model explains many pattern formation phenomena well, it is not universally applicable. Pattern formation based on this principle is largely dependent on the initial morphogen configuration, which makes it vulnerable to disturbance. On the other hand, the phenomenon of animal development is generally known to be a highly robust system. For example, hydra and planaria can regenerate a whole body from a small part, and partial regeneration is universally observed in other higher organisms. This fact suggests that an autonomous system, such as Turing's mechanism, must intervene. The most direct way to prove the existence of Turing's principle is to apply perturbations to a spatial pattern of living things and observe their dynamic reproduction process. The skin patterns of animals, as well as those of fish and amphibians, are most suitable for this purpose. In many other experimental systems, regeneration is difficult to analyse because it involves many complex phenomena, starting with wound healing [8]. In addition, regeneration occurs only in the very early stages of development. However, the skin patterns of fish and amphibians can often be regenerated in adults, and disrupting the pattern does not affect the survival of the individual [2]. Furthermore, dynamic changes in patterns can be observed without experimental manipulation. In fact, it was the change in the pattern of the emperor angelfish that first alerted experimental biologists to the existence of Turing's principle in living organisms [11]. As for the discovery of detailed molecular principles, one of the earliest was the skin pattern of the zebrafish to which many molecular genetic technologies can be applied. [12–15]

Today, Turing's principle is accepted by many experimental researchers and is used to analyse many morphogenetic phenomena [2,3]. The recent discovery that the number of fingers in mice is determined by Turing waves has made it clear that Turing's principle is involved in the formation of important structures in living organisms and has increased interest in the theory [3,16]. However, the importance of pigment patterns, especially in theoretical studies of morphogenetic principles, does not change, as there is still no system other than that of skin patterns in which dynamic changes in patterns can be observed in two dimensions. In this review, recent experimental studies using zebrafish are summarized for theoretical researchers who are new to biological pattern formation, and progress in theoretical studies based on experimental results is described.

## 2. Experimental studies

### (a) Zebrafish as the model system for studying skin pattern formation

Zebrafish are the most commonly used fish species for studying pattern formation principles. Four black and yellow stripes are present on their body and tail fins. The zebrafish is one of the model animal species in experimental biology research, and the abundance of genomic information, stocks of mutant strains and tools for genetic manipulation makes it suitable for analysing complex phenomena at the molecular level [12]. Therefore, studies on the principles of pattern formation have mainly been conducted using this species [13–15].

### (b) Autonomous regeneration of zebrafish skin patterns after disturbance

The ability of zebrafish to autonomously generate skin patterns can be demonstrated by a few simple experiments. Using a temperature-sensitive strain of the panther gene, it was found that after the pigment cells were killed to remove the pattern and then regenerated, the stripes were regenerated as well [17]. During this process, a labyrinthine pattern is created in the tail fin with the same stripe spacing but with a disordered orientation. This difference from the original pattern indicates that the stripes are generated autonomously, rather than that positional information is retained.

**Figure 1.**
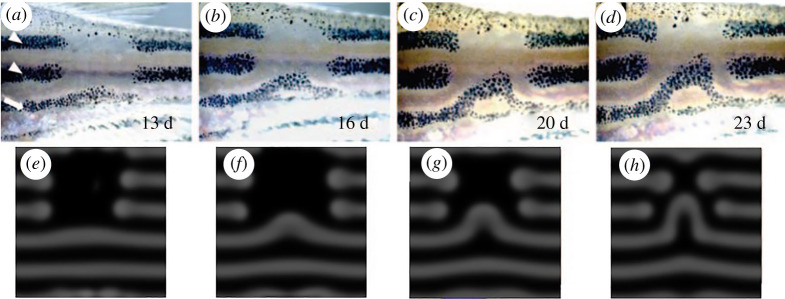
Zebrafish skin patterns with dynamic properties specific to Turing patterns. (*a–d*) Pattern rearrangement induced by partial ablation of the stripes in zebrafish. (*e–h*) Computer simulation of the reaction–diffusion model. Fish images were captured at 13, 16, 20 and 23 days after the ablation of the melanophores in the dorsalmost and central black stripes (indicated by white arrowheads). For details, see [18]. (Online version in colour.)

Yamaguchi *et al*. [18] used a laser to observe the movement of the surrounding stripes induced by erasing part of the pattern. As shown in figure 1*a–d*, the more ventral stripes filled the empty space by sliding sideways. It is noteworthy that in the generated pattern, normal spacing between the stripes was maintained. This interesting dynamic can be accurately predicted by Turing's reaction–diffusion model (figure 1*e*–*h*). These experimental results strongly suggest that a principle homologous to Turing's model is at work in the pattern formation of zebrafish.

### (c) Skin patterns are created by interactions among two (or three) types of pigment cells

The body and fins of zebrafish contain three types of pigment cells [12], and their skin pattern is determined by the arrangement of these pigment cells. The main factor in pattern formation is the interaction between the three types of pigment cells. However, it is known that the interaction between melanophores and xanthophores is sufficient for pattern formation in the fins, because even mutants lacking iridophores produce normal patterns in the fins [13,19,20]. In addition, even mutants with a drastically reduced number of iridophores in the body produce a large spot pattern (which is a type of Turing patterns), suggesting that the role of iridophores is supplementary. Therefore, the interaction analysis for pattern formation is performed mainly with melanophores and xanthophores. The study of interactions involving iridophores is ongoing, and agent-based models (ABMs) are being used for theoretical analyses [13,14,21–24].

**Figure 2.**
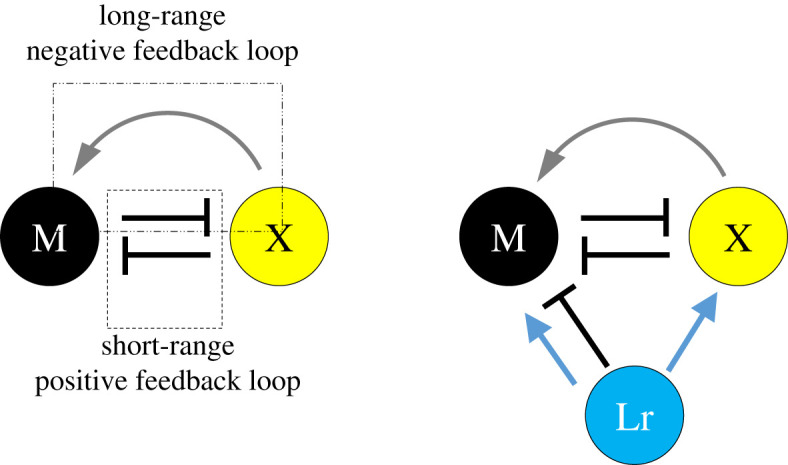
Currently proposed models of cell–cell interaction. In the fin, normal stripes can be produced even without iridophores. Therefore, the network is composed of two types of cells: melanophores and xanthophores. (*a*) Melanophore-xanthophore network. (*b*) Interaction network among the three cell types. In the body, the absence of iridophores results in an abnormal pattern; therefore, in addition to the network in (*a*), iridophores are added to the interaction. For more details and the experiments on which the model is based, see [15]. Abbreviations: Ir, iridophore; M, melanophore; X, xanthophore. (Online version in colour.)

Nakamasu *et al*. [25] investigated how melanophores and xanthophores affect each other's survival by using a laser to erase pigment cells in the middle of the formed pattern. The results are summarized in figure 2*a*. At close range, melanophores and xanthophores kill each other (thus suppressing survival) [25]. However, the opposite is true at long distances. When the laser kills all the xanthophores in a large area, cell death also occurs in the melanophores, indicating that xanthophores located at a distance are necessary for the survival of melanophores [25]. Mathematically, the network of interactions shown in figure 2*a* has similar properties to that of the original Turing's model, as it includes both activation loops working at short distances and inhibition loops working at long distances [15]. In fact, a simple ABM created using this network can form stripes from random patterns. [21–24]. A three-cell network model with iridophores is shown in figure 2*b*.

### (d) Function of cell protrusions as an alternative to molecular diffusion

**Figure 3.**
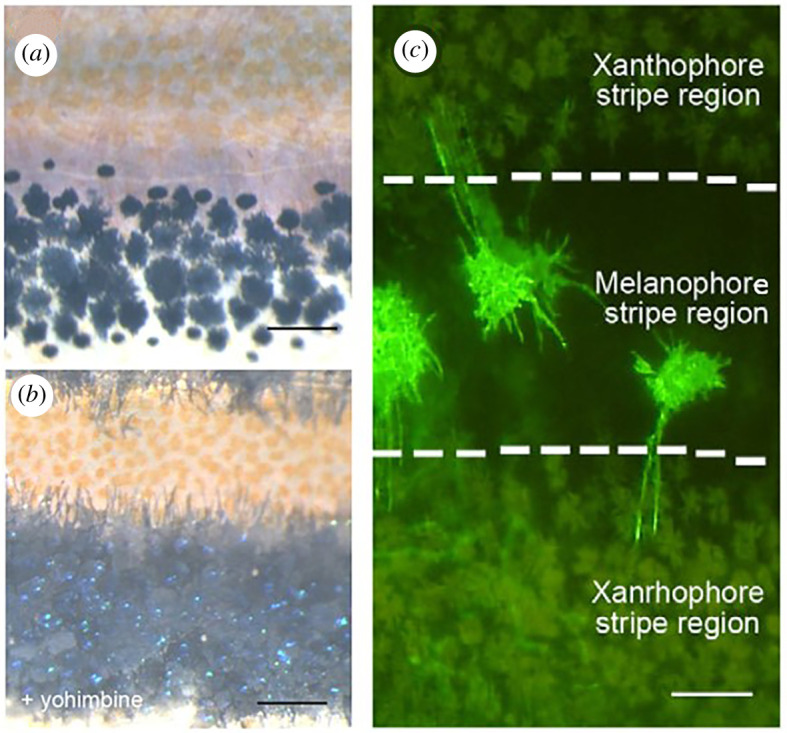
Cell protrusions of melanophores are responsible for long-distance signalling. Interactions between pigment cells are conveyed by direct action through cell protrusions rather than by diffusible signalling molecules. (*a*) Pigment cells on the body side of zebrafish. (*b*) The addition of yohimbine causes the pigment to migrate to the saccule, making the cell protrusions visible. (*c*) GFP(green-fluorescent protein) visualization of melanophore cell membrane at the centre of the black stripe. Long cell processes can be seen extending into the yellow stripe where xanthophores are located. Figures are from [28]. (Online version in colour.)

An unexpected discovery was made regarding the mechanism responsible for cell–cell interactions. When isolated melanophores and xanthophores were co-cultured *in vitro*, the cells were found to repel each other, but the signal was transmitted by short protrusion extending from the xanthophores [26,27]. Regarding distant interactions, Hamada *et al*. [28] found that long cell protrusions extending from the melanophores were involved (figure 3*a*–*c*). Interestingly, neither short- nor long-range interactions are mediated by ‘diffusion’. Therefore, strictly speaking, pattern formation does not occur by a reaction–diffusion system. However, mathematically, it can be considered as a homologous phenomenon, because the protrusions with two different lengths mimic the role of two different molecules with different diffusion coefficients in a reaction–diffusion system [15].

### (e) Genes involved in the skin pattern formation

In zebrafish, many genes involved in pattern formation have been identified, and these genes contribute to the understanding of pattern formation principles.

Although many different types of genes are involved in pattern formation, those of importance for studying the principles of pattern formation belong to the following three categories [14]:

(1) Genes affecting the development of one type of pigment cell
(2) Genes affecting stripe width and integrity, which are mainly involved in the interactions among pigment cells
(3) Genes affecting the surrounding tissue (i.e. the environment of the area where the pattern forms)

**Figure 4.**
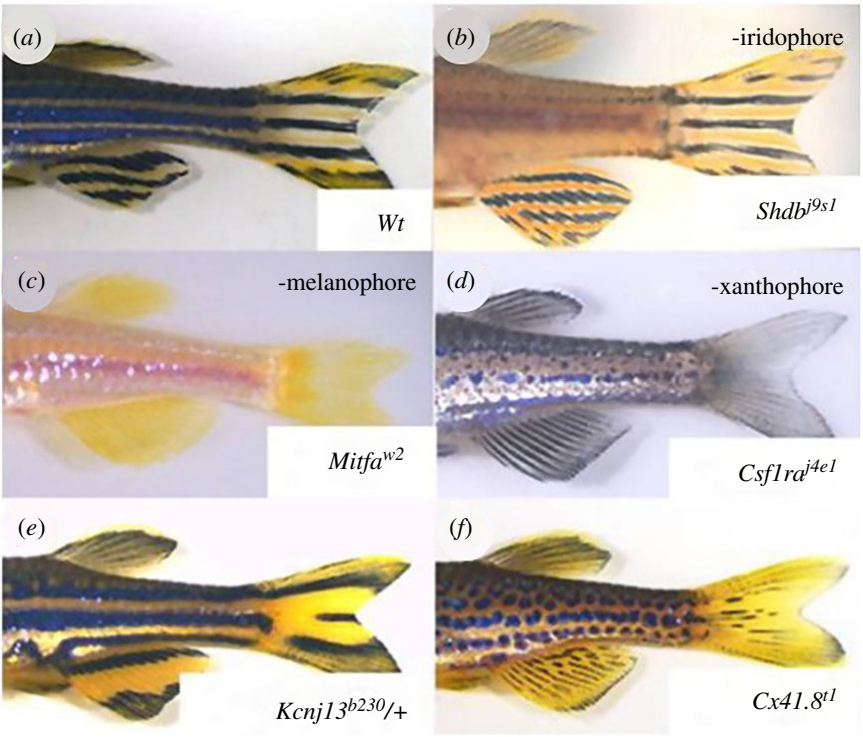
Pigmentation patterns in zebrafish mutants. (*a*) Wild-type (Wt) zebrafish exhibit normal stripe patterns in both the body and fins. (*b*) In mutants lacking iridophores (shdj9s1), normal stripes form in the fins. (*c,d*) In mutants lacking melanophores (mitfaw2) or xanthophores (csf1raj4e1), patterns are lost in both the body and fins. (*e,f*) Mutants, such as kcnj13b230 and cx41.8t1, form distinct but altered patterns. Patterns in the body and fins are similar, suggesting that essentially identical mechanisms underlie these patterns. The original mutant name for each gene is as follows: shd = shady, mitfa = nacre, csf1ra = panther, kcnj13 = jaguar/obelix, cx41.8 = leopard. For details, see [13–15]. (Online version in colour.)

The skin patterns belonging to the categories 1 and 2 are shown in figure 4*b*–*d*. When genes from category 1 are lost, a specific pigment cell is lost, and the role of that cell in pattern formation can be examined. When genes in category 2 or 3 are lost or their activities are changed, pigment cells develop normally, but the distribution pattern changes. (Figure 4*e,f*)

Recently, two unexpected insights have emerged with regard to the cellular interactions that establish pigment patterns. The first concerns the role of macrophage in the elongation of pigment cell protrusions. Parichy's group found that at the metamorphosis stage in zebrafish, macrophages contact the tips of xanthophore cell protrusions and guide the direction of elongation into melanophores. Xanthophores use these cell protrusions to facilitate the separation of melanophore from xanthophore region on the skin [29,30]. The finding could shed new light on our understanding of pattern formation phenomena, since the direction of cell elongation would influence the anisotropy of the pattern. Another finding is related to the function of gap junctions in interactions between pigment cells. Both chromophores (melanophores and xanthophores) express two types of gap junction genes, cx39.4 and cx41.8 [31–34]. The deletion of either gene results in wavy stripe or spot patterns. Watanabe & Kondo [35] have shown that artificially changing the activity of gap junctions results in various changes in skin patterns, suggesting that gap junction signalling plays a major role in skin pattern formation. However, the functions of the individual genes are not well understood, as both the chromatophores express two types of gap junctions and may also establish hetero combination gap junctions. Recently, Usui *et al*. [36] investigated the minimal conditions under which a stripe pattern could be created by expressing each gene only in melanophores or only in xanthophores, using a line in which both gap junction genes were deleted. The results showed that the expressions of cx39.4 in melanophores and cx41.8 in xanthophores are sufficient to form normal stripes. In addition, the paper showed that the cx39.4 has a polyamine dependent rectification property.

We do not discuss individual genes in detail because the purpose of this review is to provide theoretical researchers with an overview of the research to date. For more information, please see the following reviews, which list in detail the mutated genes and the resulting pattern changes [12–15].

## 3. Theoretical studies

### (a) Problems identified by experimental studies

Until now, simulations to reproduce pigment pattern formation have been mainly based on the classical reaction–diffusion model. Since the reaction–diffusion model can not only create various patterns but also accurately reproduce pattern changes, the overall mechanism of pigment cell formation in real skin exhibits a mathematical function that is very similar to that of Turing's model. However, as mentioned above, the primary processes at the cellular level include many that are not envisioned in the classical reaction–diffusion model [15]. What established the pattern is not the shading of the chemicals, but the distribution of cells that behave autonomously. Another major difference is that long-distance signalling is conveyed directly by cell protrusions rather than by molecular diffusion. Mathematical analysis results showed that if the system as a whole satisfies LALI (local activation and long inhibition) conditions [37–39], a Turing pattern would be created regardless of the type of reaction in the specific primary process. However, it does not make sense to simulate phenomena not influenced by diffusion using a mathematical model based on diffusion. The problem has been widely acknowledged since the principles at the cellular level have been elucidated, and several attempts have been made to resolve it.

### (b) Agent-based models to specifically simulate individual cell behaviour

In the case of zebrafish, the skin pattern is determined by the arrangement of several types of pigment cells and the interaction between these cells. To make the mathematical model more realistic, it is better to consider each cell as an independent entity and to express the conditions that determine the behaviour of the cells in a mathematical expression. Simulations of this idea have already become popular as ABMs, which are used to predict the impact of autonomous behaviour of consumers and other individuals on society as a whole.

**Figure 5.**
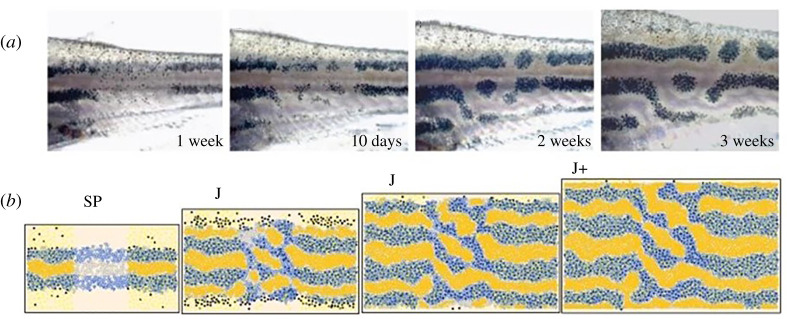
An example of pattern formation analysis by ABM. (*a*) Regeneration process after the laser destruction of pigment cells. (*b*) ABM simulation of the behaviour of the three types of pigment cells. In this study, we investigated the effect of iridophores on the robustness of stripe patterns. For more details, refer to [24]. (Online version in colour.)

Since the late 2000s, several studies have used ABMs and similar models to reproduce pigment pattern formation in zebrafish [21–24]. Volkening & Sandstede [24] defined three cell types (melanophores, xanthophores and iridophores) and their subclasses and, based on reported experimental data and speculation, they specified the behaviour of individual cells (e.g. generation, disappearance, migration and chemical production) and simulated the process of pattern formation. Their model reproduces not only the developmental process of pigment patterns in zebrafish, but also the changes that occur when disturbances such as cell removal by laser ablation are included (figure 5). They also succeeded in reproducing the differences in patterns caused by several genetic mutations [24]. This method makes it easier to compare the simulation and experiment because calculation output is the behaviour of individual pigment cells, rather than the wave pattern in the continuous field, which is the output of the classic reaction–diffusion system.

However, it should be noted that ABM is a complex system containing a large number of elementary processes. Ideally, the constants for each reaction should be determined experimentally; however, in practice, this is very difficult, which is why in the current situation, values that reproduce known phenomena are often chosen as estimates. This means that the reliability of the ABM calculation results is questionable. As might be expected, this problem is not limited to ABMs, but is common to all simulations. The value of a simulation lies not in how close the model is to reality, but in how useful the predictions are. By constructing an ABM that is appropriate for a given experimental study, it is possible to make predictions that are sufficiently reliable. Therefore, to make ABMs useful, they should be complemented with extensive experimental studies.

### (c) Cell–cell interaction transferred via cell protrusion

In most of the ABM simulations performed so far, long-range signalling has been carried out assuming a diffusive molecule, which is meant to simplify the computation, since the number of parameters increases enormously when signalling by cell protrusions is adopted in the ABM model. To study the pattern-forming ability of protrusion-based signal transduction, Vasilopoulos & Painter [40] used a simple system with a six-sided lattice of immobile cells interacting at their protrusions. They observed that even if the protrusions are not anisotropic, they can create patterns similar to those in the reaction–diffusion model by adjusting their length and density. They also observed that by adding anisotropy to the protrusions, they could create some patterns that could not be created in the two-component reaction–diffusion system. Such results could be incorporated into more detailed ABM simulations. Since the importance of cell protrusions in signal transduction has been well recognized in recent years [41], it is vital to understand the nature of the patterns created by the cell protrusions.

### (d) Comprehensive analysis of network structures that generate patterns

However, the number of factors influencing the actual pattern formation phenomenon, such as the skin pattern, is likely to be three or more. The network structure of a three-component system is much more diverse than that of a two-component system, and it would be interesting to determine what conditions must be satisfied to produce stable patterns. One such study is to exhaustively examine the pattern-forming ability of many kinds of computer-generated networks. Marcon *et al*. [42] investigated whether a stable stationary wave pattern would be produced in a three-factor reaction–diffusion system for all possible networks that could exist. The key finding of this analysis is that differences in diffusion coefficients are not essential, but rather that the properties of the network as a whole are key to stable pattern formation. For more details, please refer to [42,43].

### (e) More abstract mathematical model

Another way to resolve the difference between real phenomena and models is to make the model more abstract, so that it can be applied to any phenomenon. It is known that any mathematical model that satisfies the LALI condition will produce periodic patterns, regardless of the specific reactions assumed in the model [37–39]. Therefore, it should be possible to generate a Turing pattern by omitting the elementary processes of individual reactions and performing convolutional integration of only the conditions of LALI as a profile (=kernel) of distance and reaction. This idea itself is not unusual and was described by Murray [4]. A mathematical model using this idea is presented by Kondo [44] and available on the following webpage: https://www.fbs.osaka-u.ac.jp/labs/skondo/simulators/KernelPatternGeneraterGauss_Web/KernelPatternGeneraterGauss.html

**Figure 6.**
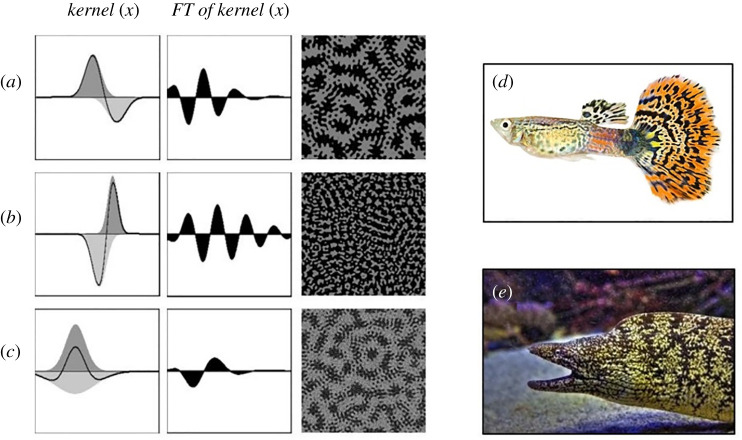
Nested patterns generated by the KT model and real fish with similar skin patterns. (*a,b,c*) Three types of kernels, their Fourier transforms and the two-dimensional patterns created by each kernel shape. All simulations used random patterns as initial conditions. (*d*) A guppy with a nested pattern on its tail fin. (*e*) Japanese moray eel. FT, Fourier transform. For more details, refer to the paper [35]. (Online version in colour.)

The abstract nature of the Kernel–Turing (KT) model is advantageous for investigating the mathematical properties of Turing patterns. If a parameter analysis is performed using a model that assumes a specific reaction, such as reaction–diffusion, the results can only be applied to the model used for the analysis. Therefore, it is impossible to know the general conditions that produce a stable Turing pattern or the general conditions that produce the difference between spots and stripes. The KT model, however, does not assume any specific reaction, but expresses the relationship between the strength of the reaction and the distance (the kernel), so these conditions can be derived. For example, according to the analysis using the KT model, to create a stable Turing pattern, the following two conditions must be satisfied: (i) the peak positions of activation and suppression must be different and (ii) the integral value of the kernel must be close to 0. Moreover, the differences in two-dimensional patterns (spots, stripes and meshes) must depend only on the integral value of the kernel. When the integral value is close to zero, a stripe (maze) pattern is produced. When the integral value is shifted positively or negatively, spots or meshed pattern is produced, respectively. For details, refer to [44]. The relationship between the simple kernel shape and the patterns produced can be easily extracted. In addition, because the Fourier transform of the kernel reveals the wavelengths that appear in advance, we can predict the patterns without actually performing a two-dimensional calculation. For example, when the Fourier transform of the kernel has multiple positive peaks, stable waves of two different wavelengths emerge (figure 6*a*–*c*). Although species with such nested patterns actually exist (figure 6*d*,*e*), it is difficult to create them with ordinary reaction–diffusion models. This proves the effectiveness of the pattern analysis using the KT model [44].

## 4. Future directions

### (a) Strides beyond the stripes of zebrafish

One of the most exciting challenges in biology is to uncover the mechanisms that give rise to enormous biodiversity. To date, research on the mechanisms of pigment pattern formation has mainly focused on the zebrafish, as it is almost the only model vertebrate that has a distinct pigment pattern [45]. However, just as studies of *Drosophila* embryogenesis did not solve all problems of developmental biology, studies on zebrafish alone will not be sufficient to reveal all the mysteries of pigment pattern diversity.

Owing to the explosive advances and popularization of sequencing technologies and the accompanying development of new analytical methods, such as single-cell RNA-seq [46,47], ancient DNA [48] and environmental DNA [49], studies on pigment pattern formation in a variety of non-model organisms are now increasing [50–53]. In this context, theoretical frameworks for morphogenesis are expected to provide insights not only into the process of pattern formation in ontogeny, but also into the driving forces behind pattern diversity and evolution.

### (b) Unravelling the enigma of pigment pattern evolution

**Figure 7.**
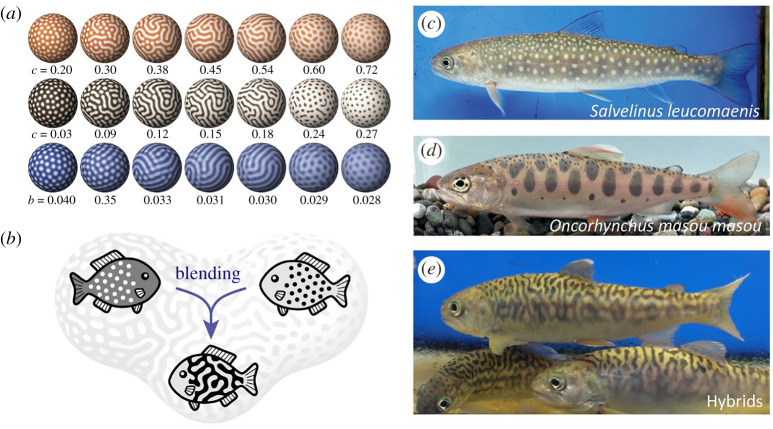
Pattern blending by hybridization. (*a*) Patterns generated by numerical simulations based on the Gierer-Meinhardt model (top), linear model (middle) and Gray-Scott model (bottom). Each colour represents the concentration of the core factor in each model (activator or autocatalytic enzyme). Lighter colours indicate higher concentrations. (*b*) The ‘pattern blending’ hypothesis. The reaction–diffusion models predict that complex patterns can be formed by pattern blending between simple motifs via hybridization. (*c*–*e*) Body patterns of salmonid fish. (*c*) White-spotted charr (*Salvelinus leucomaenis*), (*d*) masu salmon (*Oncorhynchus masou masou*) and (*e*) their artificial intergeneric hybrids. (Figures modified from [45] and [47]). (Online version in colour.)

Here, as an example of such attempts, we introduced an approach to the evolutionary question of how animal pattern diversity can arise and be maintained, which stems from predictions based on the reaction–diffusion model. As we have seen, the reaction–diffusion model and other related models can reproduce various patterns found in animal bodies. By gradually changing parameter values in an appropriate model and parameter set, one can obtain patterns that continuously shift from a spotted pattern to a reticulated (or inversely spotted) pattern (figure 7*a*).

Miyazawa *et al*. [54] focused on the point that, in the region of parameter space intermediate between these two patterns, a maze-like pattern almost always appears. They interpreted this biologically to predict that a cross between a white-spotted animal and a black-spotted animal would result in a hybrid with a maze-like pattern as an intermediate phenotype (figure 7*b*). This biologically non-trivial prediction was confirmed by artificial hybridization of living animals: all hybrids resulting from the crossing between white-spotted char and black-spotted trout indeed exhibited labyrinthine appearances (figure 7*c*–*e*) [54].

In nature, many species have labyrinthine patterns [55]. Based on the above predictions from the reaction–diffusion model, Miyazawa [56] hypothesized that species with complex labyrinthine patterns have emerged by ‘pattern blending’ through interspecific hybridization. To test this, he conducted a comparative genomic analysis of fish species in the genus *Arothron* and found that the multiple pufferfish species with labyrinthine patterns were actually hybrids derived from crosses between white-spotted and black-spotted species. Miyazawa further pointed out that such pattern blending may have contributed to the diversity of colour patterns in many fish lineages through hybrid speciation, based on the comprehensive analysis of body patterns in over 18 000 fish species and phylogenetic comparative analysis of their evolution [56]. These studies showed that the reaction–diffusion model of developmental processes can provide insights into biological diversity and evolution at the macroscopic level.

## 5. Conclusion

Although there are numerous pattern-forming phenomena, skin patterns are almost the only example where dynamic changes in patterns can be observed without experimental manipulation. This property has been a major advantage in experimental and theoretical studies and has facilitated the elucidation of the molecular and cellular principles underlying pattern formation in zebrafish skin. Conversely, such experimental results have made many researchers aware of the differences among idealized, simple classical reaction–diffusion models, and real-world phenomena, and have led to new challenges in the field of mathematical modelling such as those described above.

In recent years, Turing's model has gained wide acceptance among embryologists and is used as a working hypothesis to understand a variety of pattern formation phenomena. Most of them apply the classical reaction–diffusion model; however, in the future, differences between the idealized model and reality will become clearer. Therefore, the theoretical improvements that are currently underway in skin pattern research could facilitate the understanding of other phenomena as well. To validate newly created models, experimental systems that are easy to observe and manipulate are required, and skin patterns are one of the systems that best meets such requirements. Therefore, the importance of skin patterns as a system for studying pattern formation principles will continue to increase.

## Data accessibility

This article has no additional data.
